# Size-Dependent Variability in Flow and Viscoelastic Behavior of Levan Produced by *Gluconobacter albidus* TMW 2.1191

**DOI:** 10.3390/foods9020192

**Published:** 2020-02-14

**Authors:** Christoph S. Hundschell, Andre Braun, Daniel Wefers, Rudi F. Vogel, Frank Jakob

**Affiliations:** 1 Chair of Technical Microbiology, Technical University of Munich, Gregor-Mendel-Straße 4, 85354 Freising, Germany; rudi.vogel@wzw.tum.de; 2 Department of Food Technology and Food Material Science, Technical University of Berlin, 14195 Berlin, Germany; christoph.hundschell@tu-berlin.de; 3 Anton Paar Germany GmbH, Hellmuth-Hirth-Strasse 6, 73760 Ostfildern-Scharnhausen, Germany; andre.braun@anton-paar.com; 4 Lehrstuhl für Systemverfahrenstechnik, Technische Universität München, Gregor-Mendel-Straße 4, 85354 Freising, Germany; 5 Division of Food Chemistry, Institute of Chemistry, Martin-Luther-University Halle-Wittenberg, 06120 Halle (Saale), Germany; daniel.wefers@chemie.uni-halle.de; 6 Department of Food Chemistry and Phytochemistry, Karlsruhe Institute of Technology, 76131 Karlsruhe, Germany

**Keywords:** levan, *Gluconobacter*, exopolysaccharide, hydrocolloid, molecular weight, rheology

## Abstract

Levan is a fructan-type exopolysaccharide which is produced by many microbes from sucrose via extracellular levansucrases. The hydrocolloid properties of levan depend on its molecular weight, while it is unknown why and to what extent levan is functionally diverse depending on its size. The aim of our study was to gain deeper insight into the size-dependent functional variability of levan. For this purpose, levans of different sizes were produced using the water kefir isolate *Gluconobacter albidus* TMW 2.1191 and subsequently rheologically characterized. Three levan types could be identified, which are similarly branched, but differ significantly in their molecular size and rheological properties. The smallest levan (<10^7^ Da), produced without adjustment of the pH, exhibited Newton-like flow behavior up to a specific concentration of 25% (*w*/*v*). By contrast, larger levans (>10^8^ Da) produced at pH ≥ 4.5 were shear-thinning, and the levan produced at pH 5.0 showed a gel-like behavior at 5% (*w*/*v*). A third (intermediate) levan variant was obtained through production in buffers at pH 4.0 and exhibited the properties of a viscoelastic fluid up to concentrations of 15% (*w*/*v*). Our study reveals that the rheological properties of levan are determined by its size and polydispersity, rather than by the amount of levan used or the structural composition.

## 1. Introduction

Levan is a β-2,6-linked, water-soluble fructose polymer which can be branched at position *O*1. It is produced by bacteria and archaea via secreted or cell-wall anchored, extracellular levansucrases (EC 2.4.1.10) [1,2,3]. These enzymes use the energy of the glycosidic bond of sucrose for fructose polymerization while glucose is continuously released. Many food-grade starter cultures like *Lactobacillus spp*. or *Gluconobacter spp*. produce levans [4,5,6,7,8,9,10]. Hence, levan is a natural component of sucrose-containing fermented foods such as sourdough breads [11], kefir [12] or natto [13,14]. Besides its prebiotic and health-promoting effects as a source of dietary fiber [15], high-molecular-weight levan can improve the structural properties of foods [16,17,18,19,20] and is a component of microbial biofilms [21,22,23,24,25,26]. Its functionality as a hydrocolloid is mainly linked to its molecular weight, as high-molecular-weight levan more strongly retards bread staling [16,17]. Moreover, its size and functionality can be triggered through control of the fermentation pH during its production [20]. However, to date, little is known of why, and to what extent, high-molecular-weight levan differs from low-molecular-weight levan regarding its structural and functional properties. The aim of our study was thus to produce levans of different size distributions using the kefir isolate *Gluconobacter* (*G.*) *albidus* TMW 2.1191, which encodes one levansucrase within its genome, and to analyze the linkage types and rheological properties of the isolated levans. The obtained results are correlated to obtain new insights into the size-dependent, functional variability of levan.

## 2. Material and Methods

### 2.1. Production and Recovery of Levan at Different pH Values

*G. albidus* TMW 2.1191 isolated from water-kefir [8,20] was used for the production and recovery of levan. Cells were generally incubated in 500 mL Erlenmeyer flasks, which were filled with 50 mL liquid medium to facilitate aerobic growth on a rotary shaker. At first, *G. albidus* was grown overnight (o/n) at 200 rpm and 30 °C in a liquid NaG-medium consisting of 20 g/L sodium gluconate, 3 g/L yeast extract, 2 g/L peptone, 3 g/L glycerol, 10 g/L mannitol, 3 g/L glucose (initial pH adjusted to 6.0) until an OD_600_ in the range of 2.0–3.0 was reached. Cells were then harvested by centrifugation (7000× *g*) and resuspended in 50 mL of 0.1 M buffers (pH 3.5: citric acid/Na_2_HPO_4_; pH 4.0–5.5: Na-acetate/acetic acid; pH 6.0: Na_2_HPO_4_/NaH_2_PO_4_). For efficient levansucrase release, these buffers were supplemented with 0.1 M sucrose and incubated for 3 h at 30 °C and 200 rpm [27,28]. Afterwards, cells were separated by centrifugation (7000× *g*) and discarded. The supernatants were collected, diluted 1:1 with the same (unfermented) buffer initially used for the levansucrase release (+ 0.1 M sucrose) and statically incubated for total levan production at six different pH values (24 h, 30 °C). Finally, levan samples were dialyzed (MWCO: 3.5 kDa) against ddH_2_O (4 °C; 48 h) for removal of sugars and fructooligosaccharides <3.5 kDa, lyophilized and weighed. For fermentative levan production without pH control, *G. albidus* was cultivated for 48 h in NaG medium containing 80 g/L sucrose as the sole sugar source. After centrifugation and discarding of cells, the supernatant was treated with two volumes of chilled ethanol to precipitate the formed levan from the fermentation broth. The precipitates were collected by centrifugation (10,000× *g*, 10 min, 4 °C), re-dissolved in ddH_2_O, dialyzed against ddH_2_O (MWCO: 3.5 kDa; 4 °C; 48 h) and lyophilized. The nitrogen contents of the levan samples isolated from NaG medium and of the levans isolated from Na-acetate buffers (pH 4.0 + 5.0) were determined using the Dumas method (DUMATHERM^®^ CN, C. Gerhardt GmbH & Co KG, Deutschland). 

### 2.2. Separation and Size Determinations of Levan Fractions

The molecular weights and root mean square (rms) radii of the recovered levans were determined by asymmetric flow field-flow fractionation (AF4; Eclipse Dualtec, Wyatt Technology, Santa Barbara, CA, USA) coupled with multi-angle laser-light scattering (MALLS) (Dawn EOS, Wyatt Technology, Santa Barbara, CA, USA) analysis and UV detection (Dionex Ultimate 3000, Thermo Fisher Scientific, Waltham, MA, USA). The lyophilized levan was initially dissolved in ddH_2_O to a final concentration of 0.1 mg/mL. A quantity of 100 µL of the respective sample (10 µg) was then injected into the separation channel, equipped with a 10 kDa cellulose membrane (Nadir regenerated cellulose). Separations were performed using a detector-flow rate of 1 mL/min and a crossflow gradient of 3 to 0.1 mL/min over 15 min, followed by 15 min of a steady crossflow of 0.1 mL/min. All chromatograms were analyzed with the software ASTRA 5 (Wyatt Technologies, Santa Barbara, CA, USA) using a dn/dc value of 0.146 mL/g [20] and the Berry model integrated in the ASTRA software. The extinction coefficients (λ = 400 nm) of levans produced at different pH values were determined in 1 mL cuvettes and a Novaspec Plus spectrophotometer (Amersham plc, Little Chalfont, UK).

### 2.3. Rheological Measurements 

Prior to rheological measurements, levan samples were dissolved in ddH_2_O. Solutions with high viscosity were centrifuged for 1 min at 1000 g to remove air bubbles. Steady and dynamic rheological measurements were carried out with a Physica MCR 501 rheometer (Anton Paar, Graz, Austria) at a constant temperature of 20 °C. A double-gap geometry (DG26.7-SS, Anton Paar, Graz, Austria) was used for measurements at low viscosities (levan produced in buffers with a pH ≤ 4, levan produced in buffer with a pH > 4 and ≤ 5% (*w*/*v*), levan isolated from NaG-medium ≤ 10% (*w*/*v*)). A cone-plate geometry (CP 50–1, Anton Paar, Graz, Austria) was used for measurements above these concentrations. Steady-shear rheological data for each levan were obtained at a concentration of 5% (*w*/*v*). In addition, the viscosity curves of levan produced in buffers (pH 4.0, 5.0 and 6.0) and levan isolated from NaG-medium were recorded in the concentration range between 1–10% (*w*/*v*) and 1–25% (*w*/*v*), respectively. The viscosity curves were measured from 0.1 s^−1^ to 1000 s^−1^ followed by a reverse sequence from 1000 s^−1^ to 0.1 s^−1^. Five viscosity values were recorded per order of magnitude over a period of 10 s using a logarithmic scale. In order to determine the viscoelastic properties of levan produced at pH 4.0, 5.0 and 6.0, dynamic-rheological measurements were carried out at a concentration of 10% (*w*/*v*). In addition, a concentration of 5% (*w*/*v*) was measured for levan pH 5.0 and a concentration of 15% (*w*/*v*) for levan pH 4.0. Strain-sweep tests (stress range 0.1–100%) were performed at an angular frequency of 1 rad/s to determine the linear viscoelastic (LVE) region at 10% (*w*/*v*). Frequency-sweep tests (angular frequency 0.1–100 rad/s) were carried out in the LVE regime, at constant strain (1.0%). All rheological measurements were carried out in triplicate. 

### 2.4. Determination of the Degree of Branching

Methylation analysis was carried out as described by Fels et al. [12], with some modifications. Briefly, levans were methylated in DMSO by using powdered sodium hydroxide and methyl iodide. Subsequently, methylated polysaccharides were recovered by extraction with dichloromethane. Due to the very low stability of fructans under acidic conditions, modified hydrolysis conditions (1 M trifluoroacetic acid, 70 °C, 30 min) were used [29]. After reduction with sodium borodeuteride and acetylation with acetic anhydride, the resulting partially-methylated alditol acetates were identified by GC–MS and semi-quantitatively determined by GC-FID. The FID response factors for terminal glucose, 1,3-linked glucose, and 1,3,6-linked glucose units described by Sweet et al. [30] were used for terminal fructose, 2,6-linked fructose, and 1,2,6-linked fructose units. NMR spectroscopy was carried out on an Ascend 500 MHz NMR spectrometer after dissolving the levans in D_2_O.

## 3. Results

### 3.1. Amounts and Sizes of the Produced Levans

**Figure 1:**
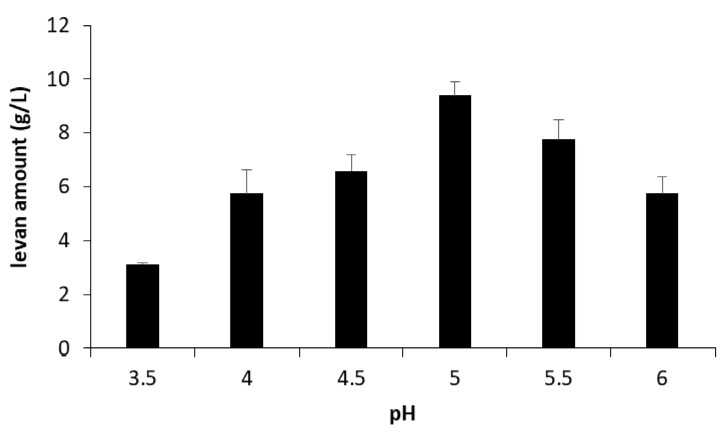
Levan amounts produced at different pH conditions in buffers. *G. albidus* TMW 2.1191 was first cultivated for 3 h at 30 °C in six buffers (pH 3.5–6.0), which contained 0.1 M sucrose, respectively, for release of its levansucrase (accession number: AQS91558) according to Jakob (2014) [26]. The obtained cell-free levan and enzyme-containing supernatants were then diluted 1:1 with the same buffer initially used for levansucrase release for the total production of levan at different pH (30 °C, 24 h).

**Figure 2:**
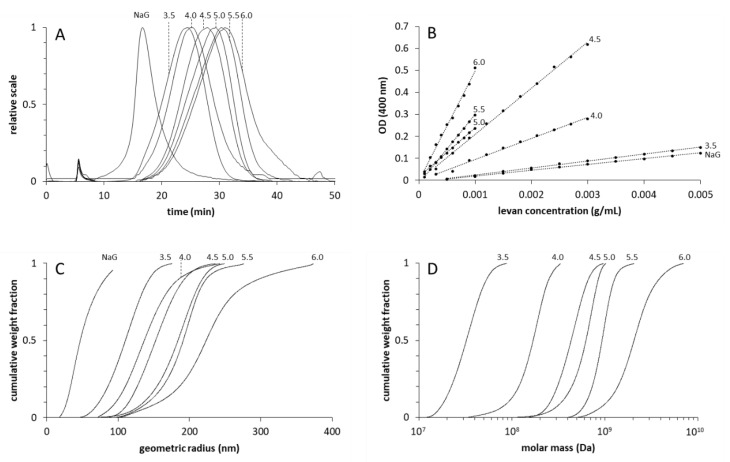
Elution profiles (light-scattering detector 11, 90°) (**A**), extinction coefficients at λ = 400 nm (**B**), cumulative distributions of geometric radii (**C**) and of molar masses (**D**) of levans produced at different pH (3.5–6.0) in buffers and in NaG medium.

Different amounts of levan were produced in buffers of different initial pH (Figure 1). The maximum levan amount could be recovered at pH 5.0. At pH 3.5, a significant decrease in levan production could be detected. Upon the growth of *G. albidus* TMW 2.1191 in NaG medium containing 80 g/L sucrose as the sole sugar source, 31.4 ± 2.3 g/L of levan could be isolated from the fermentation broth. The isolated levans were further subjected to AF4-MALLS-UV analysis (Figure 2). The lower the pH of the used buffers, the earlier the levan fractions eluted (Figure 2A). The levan isolated from NaG medium eluted distinctly earlier than the other levan samples. The molar masses and geometric radii of the levans were higher if the levan fractions eluted later, revealing an increasing hydrodynamic volume of the levan molecules with increasing molecular weight (Figure 2C,D). Moreover, the extinctions (λ = 400 nm) of the levans increased with rising production pH and molecule size (Figure 2B). The molar mass distribution of the levan isolated from NaG medium could not be evaluated as no usable UV concentration signals were obtained, even if ten-times the amount of levan (100 µg) had been injected into the separation channel. 

### 3.2. Rheological and Structural Properties of the Produced Levans

**Figure 3:**
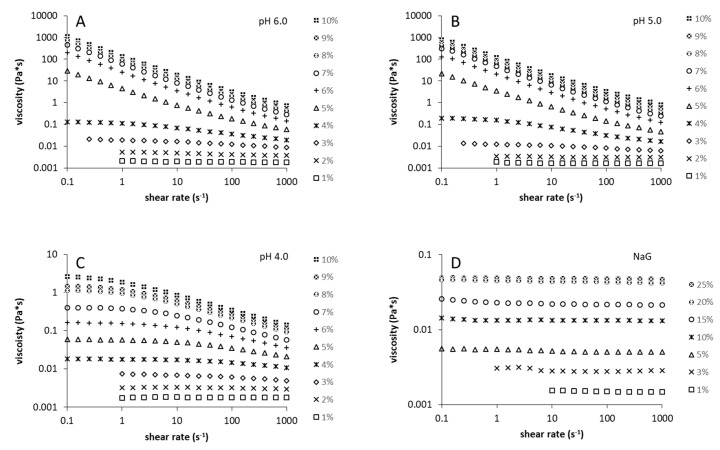
Concentration-dependent viscosity curves of levans produced at pH 6.0 (**A**), pH 5.0 (**B**), pH 4.0 (**C**) and in NaG medium (**D**) recorded at 20 °C, respectively. Noisy data obtained at low shear rates and concentrations are not depicted.

**Figure 4:**
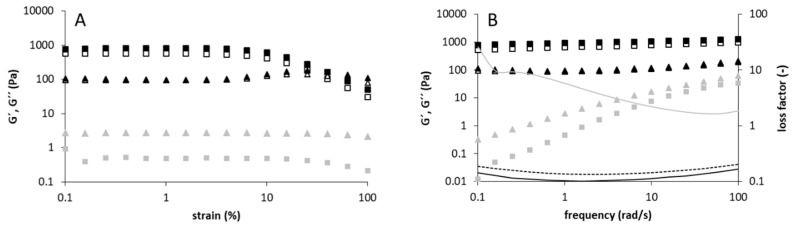
Strain-sweep test (**A**) and frequency-sweep test (**B**) of levans produced at pH 6.0 (black), pH 5.0 (white) and pH 4.0 (grey). Data were recorded at 20 °C applying a specific concentration of 10% (*w*/*v*). G’: square, G’’: triangles. Lines represent the loss factor (tan δ = G’’/G’) of levans produced at pH 6.0 (solid, black), pH 5.0 (dotted, black) and pH 4.0 (solid, grey).

At first, concentration-dependent viscosity curves were recorded for the levans produced at pH 4.0, 5.0, 6.0, and the levan isolated from NaG-medium (Figure 3). A shear-thinning behavior was observed for levans pH 4.0/5.0/6.0 at specific concentrations ≥ 3% (*w*/*v*) (Figure 3A–C). Levans produced at pH 5.0 and pH 6.0 showed almost identical viscosity-curve profiles with a sharp increase in viscosity and more pronounced shear-thinning behavior between 4% (*w*/*v*) and 5% (*w*/*v*). The levan produced at pH 4.0 exhibited a significantly lower viscosity that increased more evenly with the polymer concentration. While the zero shear viscosities of levans pH 5.0/6.0 at polymer concentrations of 4% (*w*/*v*) were ~ 10 times higher than that of levan pH 4.0 (Figure 3A–C), the viscosity of levans pH 5.0/6.0 (shear rate 0.1 1/s) was ~100 times and ~1000 times higher at polymer concentrations of 5% (*w*/*v*) and 6% (*w/v*), respectively. On the contrary, no shear-thinning behavior and no distinct viscosity increase could be detected for the levan isolated from NaG medium, even if a specific concentration of 25% (*w*/*v*) had been applied (Figure 3D). As the levan produced at pH 4.0 differed distinctly regarding its viscosity and flow behavior from levans produced at pH ≥ 4.5 (Figure 3A–C), oscillatory-shear experiments were additionally performed (Figure 4). 

The strain-sweep test (Figure 4A) revealed a fluid-like behavior (G’’ > G’) for levan pH 4.0 (10% *w*/*v*) while a gel-like behavior (G’’ < G’) was observed for levans pH 5.0/6.0 (10% *w*/*v*). Furthermore, levan pH 4.0 showed a continuous decrease of G’ and G’’ (strain thinning) and no crossover of both moduli after reaching the limit of the LVE region, while G’’ of levans pH 5.0/6.0 showed a local maximum before both moduli started to decrease (weak-strain overshoot) and finally, crossover. The strain-thinning behavior of levan pH 4.0 is commonly observed for polymer solutions, and is due to the alignment of polymer chains in terms of their flow direction at high strain [31]. The weak-strain overshoot of levans pH 5.0/6.0 could be explained by the variation of formation and destruction of the network junctions with increasing strain. An increased connectivity of the network up to a certain strain leads to an increased dissipation (maximum of G’’), while an increased network disruption at higher strains causes the decrease and the crossover of G’ and G’’, which indicates a shear-induced phase-transition from gel-like to fluid-like [32]. 

Levans pH 5.0/6.0 exhibited a gel-like behavior at a concentration of 10% (*w*/*v*) with G’’ < G’, a slight frequency dependence of both modules and a loss factor < 0.2 over the tested frequency range (Figure 4B). At 5% (*w*/*v*), levan pH 5.0 also shows a gel-like behavior (Figure S1), but both moduli and the loss factor (0.19–0.44) were more dependent on the frequency at 5%. The levan produced at a pH of 4.0 showed G’’ > G’ and both modules increased with increasing frequency at a concentration of 10% (*w*/*v*) (Figure 4B) and 15 % (*w*/*v*) (Figure S1). The loss factor of levan pH 4.0 (10% *w*/*v*) decreased from 26 to 1.65 with rising frequency (Figure 4A). Therefore, levan pH 4.0 exhibits the properties of a viscoelastic fluid at 10% (*w*/*v*) in the measured frequency range. To obtain further information about possible structural differences among the rheologically different levans, levans pH 4.0/5.0/NaG were investigated by two-dimension NMR spectroscopy and methylation analysis (Table 1). The NMR spectra were very similar for all samples and confirmed the presence of levans (data not shown). Methylation analysis allowed for the detection of 2,6-linked and 1,2,6-linked fructose units and thus linear and branched backbone units in all levan samples. The portion of terminal-fructose units was rather high (8.1–13.9%), however, this may be the result of some lowly abundant low-molecular weight fractions (which were not removed due to interactions with the high-molecular-weight polysaccharides) or modification of levans during the course of the analysis (e.g., partial hydrolysis during sonication). Nevertheless, the relative abundance of 1,2,6-linked fructose units indicates that the levans show a rather low degree of branching. In addition, the ratio between 2,6- and 1,2,6-linked fructose units (26–27) was almost constant for all samples; therefore, the structural analyses suggest that the all levans have a similar structural composition. 

Moreover, the nitrogen content of the structurally analyzed levans (Table 1) was checked using a DUMAS analyzer. The nitrogen content of the levan isolated from NaG medium was slightly higher (0.053%) than that of levans pH 4.0/5.0 (0.010–0.016%) recovered from sodium acetate buffers. 

## 4. Discussion

The yield, size and functional properties of levan from *G. albidus* TMW 2.1191 can be influenced by modulation of the environmental pH, as demonstrated in the present study and a previous study [20]. Similar findings were obtained for the dextran produced by *Lactobacillus hordei* TMW 1.1822 [33]. The environmental pH is, therefore, decisive to trigger the properties of uncharged, water-soluble high-molecular-weight exopolysaccharides produced by glucansucrases or fructansucrases [34]. However, it is yet unknown to what extent the size of levan influences its rheological properties. It was previously shown that levans produced by *G. albidus* TMW 2.1191 without external pH control are comparatively smaller in size at prolonged fermentation times, which might be due to (a combination of) continuous acidic hydrolysis, the intrinsic β-fructosidase activities of levansucrases or possibly additionally expressed β-fructosidases [10,20,35,36]. Accordingly, in the present study, the levan recovered from NaG medium after 48 h of incubation exhibited the lowest hydrodynamic volume/radius, at which an elevated nitrogen content was determined for this levan in comparison to the levans produced in Na-acetate buffers (pH 4.0 and 5.0). The presented approach for levan production in buffers could thus be used and further optimized for the recovery of considerable amounts of pure levans exhibiting different sizes and properties.

A Newton-like flow behavior was observed for the levan produced in NaG medium up to a specific concentration of 25% (*w*/*v*) (Figure 3D). A similar concentration-dependent flow behavior of levan was found by Kasapis et al. [37] and Arvidson et al. [38] for levans produced by *Pseudomonas syringae* and *Bacillus species*, respectively. In their studies, the levans also showed Newtonian flow behavior up to a concentration of 20% or 30%.

Interestingly, some changes to the rheological properties were detected for levans produced at pH ≥ 4.0. At concentrations ≥ 3% (*w*/*v*), these levans showed shear-thinning behavior and a more pronounced increase in viscosity with concentration. Both phenomena are more distinct for levans produced at pH ≥ 4.5. Furthermore, levan pH 4.0 behaved like a viscoelastic liquid, while levans produced at pH 5.0/6.0 showed a gel-like behavior in oscillatory-shear experiments (Figure 4). In the study of Benigar et al. [22], the onset of shear thinning was found between 1.0% and 4.0% for three levans of different microbiological origin. In addition, they observed an increasing dependence of viscosity and of shear-thinning behavior on concentration with increasing polymer size. A gel-like behavior comparable to that of levan from *G. albidus*, produced at pH 5.0/6.0, could also be found for high-molecular-weight levans from *Erwinia amylovora* [39] and *Brenneria species EniD312* [40].

These results suggest that levan molecules produced by *Gluconobacter* and other bacteria can interact with each other and build up intermolecular networks if a critical molecular size and a critical polymer concentration is exceeded. These networks may consist of highly entangled high-molecular-weight levan chains. In solutions of levan with low molecular size, the number of entanglement points between different levan chains are likely insufficient to stabilize the physical network. Therefore, these levans (pH 3.5, NaG) cannot form a physical network and show Newtonian-like flow behavior even at high concentrations. The levan produced at pH 4.0 exhibiting viscoelastic properties could be considered as an intermediate between low-viscosifying (pH 3.5, NaG) and gel-like levan (pH ≥ 4.5) and might have been composed of some, but too few, levan molecules exhibiting the critical chain length for network formation. Therefore, the homogeneity and polydispersity of a levan fraction also contributes to its functionality. The results from the structural analyses further suggested that the degree of branching was not a decisive factor for the rheological properties in the case of the analyzed levans.

A second model, which might explain the size-dependent rheological behavior of levan, is based on the secondary structure of levan in aqueous solution. With increasing molecular weight, levan transforms from a random coil into a compact spherical molecule, which is increasingly compact/densely packed and turbidity-forming [17,41]. Therefore, Jakob et al. [17] suggested that levan above a certain molecular weight might have the properties of a nanogel particle. Using this comparison, the viscoelastic behavior of levan of a certain size (pH ≥ 4.0) could be explained as the interaction of soft spheres forming a colloidal network [42]. 

## 5. Conclusions

Our study reveals that the rheological properties of levan are determined by its size and polydispersity, rather than by the amount of levan used or the structural composition. The pH used during the production of levan can be adjusted to control its size and composition. These findings are a key step towards biotechnological exploitation of levan and may help to better understand its functionality in complex (food) matrices and microbial biofilms. In order to gain further insight into the dependence of the functional and rheological properties on its polymer size, the structural degradation of high-molecular-weight levan at high shear rates could be determined to study thixotropy and thus, its viscoelastic behavior at longer time scales. 

## Figures and Tables

**Table 1:** Glycosidic linkages (mol%) of levans produced by *G. albidus* TMW 2.1191 at pH 4.0, pH 5.0, and in NaG medium, as determined by methylation analysis.

Glycosidic Linkage	pH 4.0	pH 5.0	NaG
t-Fru*f*	12.3 ± 3.4	13.9 ± 1.0	8.1 ± 0.4
2,6-Fru*f*	84.6 ± 3.1	83.1 ± 1.1	88.5 ± 0.3
1,2,6-Fru*f*	3.1 ± 0.3	3.1 ± 0.1	3.4 ± 0.1

## Supplementary Materials

The following are available online at https://www.mdpi.com/2304-8158/9/2/192/s1, Figure S1: Frequency-sweep test of levans produced at pH 5.0 (white) and pH 4.0 (grey). Data were recorded at 20 °C applying a specific concentration of 5% (*w/v*) (pH 5.0) and 15% (*w/v*) (pH 4.0). G’: square, G’’: triangles.
