# Supplementary material for: Size-Dependent Variability in Flow and Viscoelastic Behavior of Levan Produced by Gluconobacter albidus TMW 2.1191
Source: Foods. 2020 Feb 14;9(2):192. doi: 10.3390/foods9020192 (PMC7073539; doi:10.3390/foods9020192)
Supplement: Supplementary file 1 [file foods-09-00192-s001.zip › foods-677153-supplementary.pptx]

## Slide 1
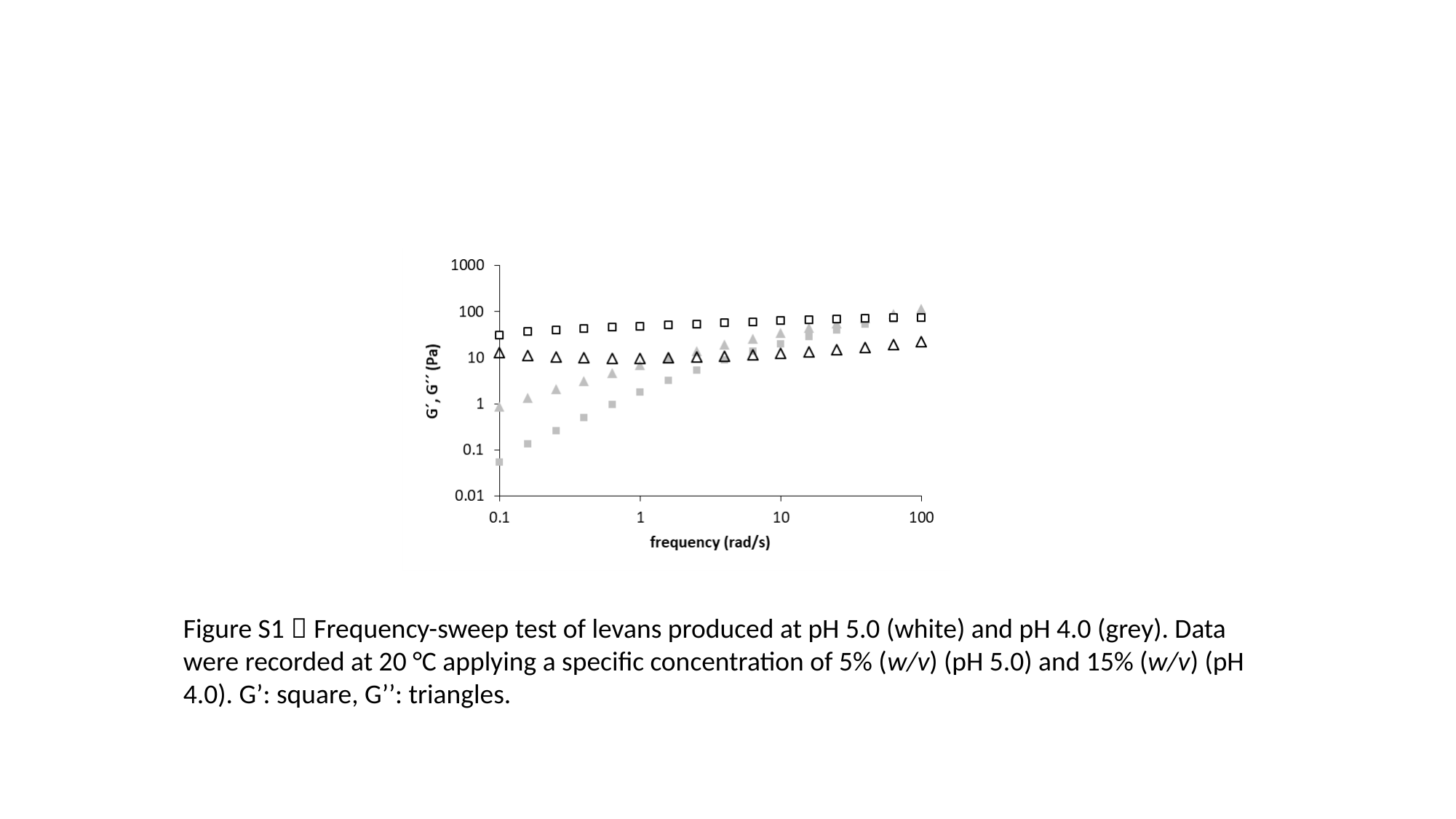

Figure S1：Frequency-sweep test of levans produced at pH 5.0 (white) and pH 4.0 (grey). Data were recorded at 20 °C applying a specific concentration of 5% (w/v) (pH 5.0) and 15% (w/v) (pH 4.0). G’: square, G’’: triangles.
